# Impact of Combined Antiretroviral Therapy on Metabolic Syndrome Components in Adult People Living with HIV: A Literature Review

**DOI:** 10.3390/v14010122

**Published:** 2022-01-11

**Authors:** Mariusz Sapuła, Magdalena Suchacz, Andrzej Załęski, Alicja Wiercińska-Drapało

**Affiliations:** Department of Infectious and Tropical Diseases and Hepatology, Medical University of Warsaw, 01-201 Warsaw, Poland; magdalena.suchacz@wum.edu.pl (M.S.); andrzejzaleski84@wp.pl (A.Z.); awiercinska@gmail.com (A.W.-D.)

**Keywords:** antiretroviral therapy, ART, HIV, metabolic syndrome, weight gain, obesity, review

## Abstract

The development of metabolic derangements as a result of HIV treatment has been an important area of research since the introduction of zidovudine in the 1980’s. Antiretroviral therapy has intensely evolved in the last three decades, with new drugs gradually incorporated into everyday clinical practice. With the life expectancy of people living with HIV rapidly approaching that of their HIV-negative counterparts, the influence of these antiretrovirals on the development of the components of the metabolic syndrome remains of major interest to clinicians and their patients. In this review, we aimed to discuss the impact of cART on components of the metabolic syndrome, i.e., weight, plasma lipid levels, plasma glucose levels, and blood pressure, describing the influence of cART classes and of individual antiretrovirals. We also aimed to outline the limitations of the research conducted to date and the remaining knowledge gaps in this area.

## 1. Introduction

The metabolic syndrome is a cluster of co-occurring conditions that serves a useful framework for identifying individuals with increased cardiovascular risk [1,2]. Many definitions of the metabolic syndrome exist in the literature, but most are dependent on the presence of central obesity, an atherogenic lipid profile, hyperglycemia mediated by insulin resistance, and hypertension [1]. One recognized definition is that of Alberti et al. [2], where an individual is classified as having the syndrome if at least three out of following criteria are fulfilled: (1) increased waist circumference with population-specific cutoffs; (2) plasma triglyceride ≥150 mg/dL (1.7 mmol/L) or on triglyceride-lowering medication; (3) plasma HDL-cholesterol <40 mg/dL (1.0 mmol/L) in males, <50 mg/dL (1.3 mmol/L) in females or on HDL-cholesterol-increasing medication; (4) systolic blood pressure ≥130 mmHg or diastolic blood pressure ≥85 mmHg or on blood pressure-lowering medication; and (5) plasma fasting glucose ≥100 mg/dL or on glucose-lowering medication.

The presence of the metabolic syndrome is considered to increase diabetes type II risk and cardiovascular risk although there is some controversy whether this is a result of the presence of the syndrome itself or just the effect of its individual components [3,4,5,6]. Other conditions were also epidemiologically linked to this syndrome, including hyperuricemia [7], polycystic ovary syndrome [8], obstructive sleep apnea [9], chronic kidney disease [10], non-alcoholic fatty liver disease [11], some forms of cancer [5], and most recently, mortality in COVID-19 [12], but for many of these associations, either a direct causal link and its direction in regards to the metabolic syndrome is yet to be proven. Known risk factors for the development of the metabolic syndrome include advanced age, certain ethnicities, other genetic factors, lifestyle factors (diet and sedentary lifestyle), some types of medication, and comorbidities [13,14,15,16,17,18]. A complex relationship exists for sex, where in younger age groups, the syndrome is slightly more prevalent in males, whereas in older age groups, the reverse is true [19].

The global prevalence of the metabolic syndrome in people living with HIV (PLWH) is estimated at 16.7–31.3%, depending on the definition of the syndrome [20]. There is some evidence that the prevalence of the metabolic syndrome in PLWH is increasing, mirroring epidemiological trends in the general population and possibly owing to the ever increasing life expectancy of PLWH [20,21,22,23,24].

The ways in which HIV infection may play a role in promoting the development of the metabolic syndrome are not fully understood. Some of the proposed points of pathophysiological convergence of the two conditions include dysregulation of the immune system with tilting towards the pro-inflammatory state, bacterial translocation, endothelial dysfunction, adipocyte dysfunction, and activation of the renin-angiotensin-aldosterone-system [25]. Fortunately, early initiation of antiretroviral therapy has been shown to dampen the immune activation seen with HIV [26,27].

Since the introduction of zidovudine in 1980s [28], antiretrovirals and later combined antiretroviral therapy (cART) have been associated with lipodystrophy as well as a deleterious effect on lipid and glucose metabolism. cART has intensely evolved in the last three decades, with new antiretroviral drugs gradually incorporated into everyday clinical practice. Besides better tolerability and safety, their adverse effect profile shifted from the typical lipodystrophy phenotype to weight gain that is more typical of the general population. The influence of cART on components of the metabolic syndrome remains of major interest to clinicians and their patients [29,30].

In this review, we aimed to summarize the current knowledge on the effect of cART on the development of the main components of the metabolic syndrome.

## 2. Obesity

### 2.1. cART and Weight Gain

In the era of effective, well-tolerated cART, overweight and obesity have become prevalent in PLWH [31,32,33] although some studies suggest the prevalence of obesity is comparable or only slightly higher than in the general population [31,34].

PLWH generally increase their body mass after starting cART, and most of this weight gain is observed in the first year of therapy [35,36,37]. The mean weight gain in this period is roughly 3–7 kg [35,36,38] or 1.6 kg/m^2^ by BMI [39], and more weight gain is to be expected in patients with lower CD4^+^ cell counts or with a higher baseline HIV viral load [32,34,35,36,38,40,41,42,43] as well as after successful management of some opportunistic conditions [44]. It is difficult to definitely conclude to what extent this is due to the medication itself, improvement of nutritional status after the suppression of HIV replication (a “return-to-health” effect), or a different biological mechanism altogether.

There is evidence that women may be a particularly vulnerable group to cART-associated weight gain [33,34,40,41,42] although some studies failed to show such associations [32,37,43,44]. Similarly, there is conflicting evidence on the role of ethnicity, with some but not all studies suggesting that black individuals are at an increased risk [34,36,42].

Switching cART can also result in body mass change. In an analysis of 12 switch studies, Erlandson et al. [37] found that PLWH gained weight regardless of whether they received a new regimen or were randomized to the comparator group. The difference between weight gain in these switch studies between stay-on and switch groups were generally in the range of 0.5–1.5 kg at week 48.

### 2.2. Class Effects and Particular Antiretrovirals

Increased weight gain in the first years of cART was associated with INSTI use in a large, well-designed cohort from North America [35]. The largest weight gain was observed in dolutegravir (DTG) recipients and moderate weight gain in raltegravir (RAL) recipients, whereas weight gain on elvitegravir boosted with cobicistat (EVG/c) was comparable to that on non-nucleoside reverse transcriptase inhibitors (NNRTIs). The cohort did not include patients on bictegravir (BIC) [35]. Similarly, in a pooled analysis of eight phase-3 clinical trials on treatment-naïve patients by Sax et al. [36], greater weight gain after initiation of cART was seen with the use of INSTIs, among which DTG and BIC were associated with greater weight gain than EVG/c. In the ACTG A5257 trial with treatment-naïve patients, there was a bigger waist circumference gain for individuals receiving RAL than for those receiving darunavir boosted with ritonavir (DRV/r), but no difference was noted for RAL vs. atazanavir boosted with ritonavir (ATV/r) [42].

In switch studies, switching from EVG/c to either DTG or BIC was associated with increased weight gain compared to staying on EVG/c, and switching from efavirenz (EFV) to EVG/c was associated with increased weight gain. No differences in weight gain were seen when switching from DTG or from a boosted protease inhibitor (PI) to BIC [37].

Cabotegravir (CAB) is the first injectable antiretroviral approved for the treatment of HIV in combination with rilpivirine. In the FLAIR study, there was no difference in weight gain between patients on injectable CAB/RPV and oral DTG/ABC/3TC [45]. No real-life data are available for CAB.

INSTIs were implicated as risk factors for weight gain in other observational studies as well [41,46], with very few reports reporting no association [47].

Apart from the above-cited comparisons of PIs to INSTIs, there are also some data on how PIs compare to NNRTIs. PIs have been associated with increased weight gain when compared to NNRTIs in a cohort from North America [35]. More weight gain on PIs than on NNRTIs was also seen in the ACTG A5257 trial, with no difference between DRV/r and ATV/r in terms of weight gain [42]. In the A5224s study, more weight gain upon initiation of cART was seen with ATV/r than with EFV [48]. Some pre-2010s data implicated PI use with weight gain as well [32,34].

Among NNRTIs, the use of rilpivirine (RPV) was associated with more weight gain than the use of EFV in treatment-naïve patients [36] and with more weight gain than EVG/c in switch studies [37]. RPV should be taken with a meal to aid its absorption, and thus, Sax et al. theorized [36] that this could lead to patients inadvertently increasing their daily caloric intake in order to comply with their physicians’ recommendations; more real-life data are needed in order to support this hypothesis.

Doravirine (DOR) is a comparatively new NNRTI first approved in 2018. In clinical trials in treatment-naïve patients, DOR was associated with a mean weight gain of 1.7 kg at week 48, which was comparable to DRV/r and slightly higher than with EFV. At week 96, there were no differences in significant (>10% body weight) weight gain or percentage of patients with BMI class increase (e.g., from underweight to normal weight, from normal weight to overweight, etc.) between regimens containing DOR, EFV, or DRV/r [43]. Similar to CAB, no real-life data are available for DOR.

There is data on the influence of nucleoside/nucleotide reverse transcriptase inhibitors (NRTIs) on changes in body mass as well. In the A5224s study, weight gain upon initiation of cART was not dependent on whether the tenofovir disoproxil/emtricitabine (TDF/FTC) or abacavir/lamivudine (ABC/3TC) backbone was used [48]. In the GEMINI-1 and GEMINI-2 trial, weight gain was comparable between DTG/3TC and DTG/TDF/FTC [49]. A large observational study failed to find a difference in weight gain between TDF and zidovudine (ZDV) recipients [41].

Tenofovir alafenamide (TAF) has been implicated in weight gain in both observational and clinical studies [46]. In Sax et al.’s analysis of clinical trials [36], greater weight gain after initiation of cART was seen with TAF use than with other NRTIs. In switch studies, switching from ABC or TDF to TAF was also associated with more weight gain than remaining on the baseline regimen [37]. The switch from TDF to TAF was associated with a weight gain of 2–4.5 kg in the first nine months after the switch in one cohort study, with a subsequent tendency for the weight to plateau [50].

### 2.3. The Consequences of cART-Associated Weight Gain

There is some controversy about whether cART-associated weight gain increases cardiovascular risk. In the above-mentioned analysis by Sax et al. [36], significant (≥ 10% body weight) weight gain at 96 weeks from cART initiation was associated with lower HDL levels and a higher total-cholesterol-to-HDL ratio but not with a difference in LDL levels, fasting glucose levels, or blood pressure. In the D:A:D cohort, an increase in BMI in the first year of cART correlated with the risk of subsequent development of diabetes, but its association with CV morbidity was more complex—an increase in BMI correlated with subsequent CVD risk only in the normo-weight group (BMI 18.5–25) and not in underweight, overweight, or obese individuals. There was no correlation between BMI change and CVD risk in the whole cohort [51]. Similarly, there was no association between cART-associated weight gain and increase in incident diabetes in a study by Herrin et al. [52] although a low percentage of female participants (<5%) makes the study difficult to extrapolate to the female population.

In an analysis of switch studies, Erlandson et al. [37] compared serum lipid levels, fasting glucose, and blood pressure between patients with at least 10% body weight gain with and patients with less than 10% body weight gain at 48 weeks after cART switch. There were statistically significant differences between the groups, with the group with significant weight gain experiencing a median decrease of HDL cholesterol levels of 3 mg/dL (vs. no change in patient without significant weight gain), a median decrease in total cholesterol levels of 2 mg/dL (vs. an increase of 3 mg/dL), and a median increase in total-cholesterol-to-HDL ratio of 0.1 (vs. no change), which are findings of uncertain if not outright doubtful clinical relevance due to the small magnitude of the observed differences. There were no statistically significant differences in change of LDL cholesterol, triglyceride and glucose serum levels, treatment-emergent diabetes- or hyperglycemia-related adverse outcomes, as well as no differences in the change of systolic and diastolic blood pressure.

In Yuh et al.’s longitudinal study [44], gaining 10 or more pounds after one year of cART was associated with decreased five-year all-cause mortality, but only in the underweight and normal weight group. In overweight and obese individuals, there was no association between cART-associated weight gain and five-year mortality whatsoever; although, as 97% of the participants were male, these findings may be not easily generalizable to the female population.

Furthermore, cART regimens with similar weight gain can have a differential effect on other traditional risk factors. This was seen in the GEMINI-1 and GEMINI-2 clinical trials, where participants in the DTG/3TC arm and in the DTG/TDF/FTC arm had similar weight gain, yet they were different in terms of changes in lipid levels, with the DTG/3TC arm associated with a more favorable lipid profile at 48 and 96 weeks [49].

More research is needed to understand the impact of cART-associated weight gain on cardiovascular morbidity and mortality, especially in the longer term.

### 2.4. Limitations of Studies on cART-Associated Weight Gain and Gaps in Research

Antiretroviral drugs are used in combination, and thus, it is sometimes difficult to determine whether such observations are due to individual components of cART or particular combinations of drugs. Most participants in clinical trials of HIV medication have a low absolute risk of cardiovascular events due to their young age; as an example, 75% of participants of the seven clinical trials described in a review paper by Sax et al. [36] and 75% of participants of the 66 trials described in a meta-analysis by Nan et al. [53] were under the age of 45 for studies on cART-naïve patients and under the age of 50 for switch studies. Due to this and due to the relatively short follow-up of most HIV trials, some authors have indicated that studies may be underpowered to detect a difference in cardiovascular outcomes between arms [53,54]. In observational studies, on the other hand, there is the well-known additional risk of confounding factors, e.g., the degree of immune deficiency and HIV viral load, guiding both the choice of cART and influencing the end net weight gain.

In European and North American HIV studies, women frequently constitute less than 15% of participants [35,36,48], reflecting epidemiological trends in these geographical areas. This can lead some studies to potentially miss important trends in cART tolerability in women, including an increased susceptibility to weight gain with certain cART combinations. As an example, in the ACTG A5257, there was a bigger difference between waist circumference gain on RAL versus on ATV/r for women than for men, which could potentially indicate differential metabolic tolerability [42]. More research is needed in order to understand the significance of gender on cART-associated weight gain and whether it can influence clinical decision-making.

Most studies on cART-associated weight gain included absolute weight change and BMI change, with no information on alternative measures of adiposity, such as waist circumference measurement or imaging studies. It is therefore difficult to determine to what extent the observed changes in weight were determined by increases in visceral adiposity, subcutaneous adiposity, muscle mass, or by other compartments, all of which have widely different effects on metabolic health [55,56,57]. To further complicate the matter, using both anthropometric measurements and imaging can lead to conflicting results and difficulties in interpretation [58]. More research is needed in order to understand which anthropomorphic and/or imaging techniques are best suited for the prediction of the effect of cART on weight gain in CVD risk-relevant compartments.

## 3. cART and Plasma Lipid Levels

PLWH have a greater risk of dyslipidemia compared to the general population. The etiology of these metabolic disturbances is multifactorial. Firstly, HIV itself is linked with chronic inflammation and immune activation, which lead to a specific lipid phenotype characterized by decreased levels of total cholesterol (TC) and high-density lipoprotein cholesterol (HDL), increased levels of LDL cholesterol (LDL) and triglycerides (TG), as well as changes in lipid composition and function [59,60,61]. Secondly, dyslipidemia may result from personal factors of PLWH, such as age, sex, genetic factors, diet, lifestyle, etc. Finally, exposure to cART is also related to serum lipid alterations.

Various classes of antiretrovirals influence clinical lipids in a differential manner. Particularly, older NNRTIs, such as efavirenz and PIs, have been associated with a greater risk of dyslipidemia than newer agents from the same antiretroviral classes [62]. As a result, first-line HIV therapies currently recommended in the European AIDS Clinical Society (EACS) are focused on antiretroviral drugs with more favorable lipid profiles [63].

TAF has been introduced into clinical practice as a new tenofovir prodrug with the advantage of lower renal and bone toxicity because of its lower plasma concentration [64]. Unfortunately, switching from TDF to TAF also resulted in significant increases in TG, TC, LDL, and HDL cholesterol levels [65,66]. However, recently it has been shown that when TAF is switched back to TDF, lipid levels return to previous values, which may be due to the possible lipid-lowering statin-like effect of TDF [67,68].

RAL, DTG, and BIC use is consistently associated with globally improved lipid parameters both in treatment-naïve PLWH and after switch from PIs, EFV, or EVG/c, and their use is associated with decreases in LDL cholesterol and triglyceride levels and often with increases in HDL cholesterol levels. Cobicistat, a booster used with EVG and optionally with some PIs, is a CYP3A4 inhibitor with lower negative influence on lipid metabolism than ritonavir. However, EVG combined with cobicistat has a worse metabolic profile than other INSTIs, and as a result, presently, it is recommended in the EACS Guidelines as a second-line HIV therapy [63,69].

DOR is associated with a lower incidence favorable lipid profile in HIV-infected cART-naïve patients [70]. Moreover, the reductions in fasting lipids were also observed after switching to once-daily DOR/3TC/TDF in virologically suppressed HIV-infected subjects [71].

## 4. cART and Diabetes

The association of cART with diabetes mellitus (DM) is inconsistent and varies widely across primary epidemiological studies [72,73,74,75]. PI use has been associated with hyperglycemia, but data from the large studies are inconsistent [76,77,78,79].

A meta-analysis that included twenty cross-sectional studies involving more than five thousand participants in sub-Saharan Africa showed no association between use of antiretroviral therapy and DM (OR: 2.53, 95%CI: 0.87–7.35, *n* = 8, I2 = 73.8%) [80]. A different meta-analysis conducted in Africa showed no significant differences between type 2 DM (T2DM) prevalence in PLWH versus uninfected individuals (risk ratio (RR) = 1.61, 95% CI 0.62 to 4.21, *p* = 0.33) or between cART recipients versus untreated patients (RR = 1.38, 95% CI 0.66 to 2.87, *p* = 0.39); however, these results are limited by the high heterogeneity of the included studies and moderate-to-high risk of bias [81]. Another meta-analysis describing almost fourteen thousand HIV patients revealed that use of PIs was not associated with the development of DM [82].

On the other hand, data from more than twenty thousand participants from 41 observational studies concerning the proportions of DM in PLWH showed that mean fasting plasma glucose (FPG) concentrations and the odds of diabetes were significantly higher among cART-exposed patients compared to their naïve counterparts. cART was also associated with significant increases in FPG levels in studies with mean cART duration ≥18 months (pooled mean difference: 4.97 mg/dL; 95% CI, 3.10 to 6.84; 14 studies) but not in studies with mean cART duration <18 months (pooled mean difference: 4.40 mg/dL, 95% CI, −0.59 to 9.38; 7 studies). However, given the predominance of cross-sectional studies in the meta-analysis, causality between cART and diabetes could not be presumed [83]. A meta-analysis describing the relationship between HIV infection, HIV treatment, and gestational DM (GDM) showed no significant relationship between HIV infection and the development of GDM (risk ratio 0.80, 95% confidence interval (CI): 0.47–1.37, I = 0%), but exposure to PIs increased GDM risk in studies using first-generation protease inhibitors (risk ratio 2.29, 95% CI: 1.46–3.58) and studies using the strictest diagnosis criteria, which is the National Diabetes Data Group criteria for 3-h oral glucose tolerance test (risk ratio 3.81, 95% CI: 2.18–6.67) [84].

## 5. Hypertension

In observational studies, PLWH on cART have on average 4.5-mmHg higher SBP and 3.2-mmHg higher DBP than their cART-naïve counterparts, which translates into a 1.68 higher odds of having hypertension [85], with this trend visible even after stratifying for age [86]. It is not known whether this should be considered as a direct effect of cART or a phenomenon analogous to return-to-health seen with cART-associated weight gain. Compared to other components of the metabolic syndrome, less is known about the influence of particular cART classes and of particular drugs on the development of hypertension.

ZDV has been linked to hypertension in some observational studies [87,88] with conflicting results [89] or an inverse association in others [90]. An experimental study on ZDV-fed rats showed that, compared to controls, they had, among other alterations, higher blood pressure, hypertrophic myocytes, and modified vascular smooth muscle responsiveness [91], but it is difficult to determine whether this was due to a specific effect of ZDV, a class effect of NRTIs, or an effect of antiretrovirals altogether.

There is evidence that the activation of the renin-angiotensin-aldosterone system (RAAS) specifically may contribute to an increased prevalence of hypertension in PLWH [25]. Interestingly, DRV, a member of the PI group, was proven through computational methods to exhibit a dual inhibitory effect, inhibiting not only the HIV protease but also renin, a member of RAAS [92]. This was reflected in the results of one large African-based study, where significantly lower diastolic blood pressure and a non-significant trend towards lower systolic blood pressure in patients on PI-containing regiments were observed [93].

## 6. Conclusions

cART poses a unique risk factor for PLWH for the development of individual components of the metabolic syndrome, with particular drugs and classes associated with different degrees of risk in this regard. The knowledge of these different metabolic profiles of antiretrovirals can be used by clinicians to tailor cART to particular patients in order to minimize their cardiovascular morbidity and mortality. The continued surveillance of new generations of antiretrovirals in regards to their effect on metabolic health, both in clinical trials as well as in real-life cohorts, is important in order to not miss new trends in cART toxicity.

## Data Availability

Not applicable.
